# Different clinical features in Malawian outpatients presenting with COVID-19 prior to and during Omicron variant dominance: A prospective observational study

**DOI:** 10.1371/journal.pgph.0001575

**Published:** 2023-03-08

**Authors:** Marah G. Chibwana, Herbert W. Thole, Cat Anscombe, Philip M. Ashton, Edward Green, Kayla G. Barnes, Jen Cornick, Ann Turner, Desiree Witte, Sharon Nthala, Chikondi Thom, Felistas Kanyandula, Anna Ainani, Natasha Mtike, Hope Tambala, Veronica N’goma, Dorah Mwafulirwa, Erick Asima, Ben Morton, Markus Gmeiner, Zaziwe Gundah, Gift Kawalazira, Neil French, Nicholas Feasey, Robert S. Heyderman, Todd D. Swarthout, Kondwani C. Jambo

**Affiliations:** 1 Malawi-Liverpool-Wellcome Programme (MLW), Blantyre, Malawi; 2 Institute of Infection, Veterinary and Ecological Sciences, University of Liverpool, Liverpool, United Kingdom; 3 Department of Clinical Sciences, Liverpool School of Tropical Medicine, Liverpool, United Kingdom; 4 Harvard School of Public Health, Boston, MA, United States of America; 5 Broad Institute of MIT and Harvard, Cambridge, MA, United States of America; 6 University of Glasgow MRC Centre for Virus Research, Glasgow, United Kingdom; 7 Blantyre District Health Office, Ministry of Health, Blantyre, Malawi; 8 NIHR Global Health Research Unit on Mucosal Pathogens, University College London, London, United Kingdom; 9 Julius Center for Health Sciences and Primary Care, University Medical Center Utrecht, Utrecht University, Utrecht, Netherlands; 10 Kamuzu University of Health Sciences, Blantyre, Malawi; PLOS: Public Library of Science, UNITED STATES

## Abstract

The SARS-CoV-2 Omicron variant has resulted in a high number of cases, but a relatively low incidence of severe disease and deaths, compared to the pre-Omicron variants. Therefore, we assessed the differences in symptom prevalence between Omicron and pre-Omicron infections in a sub-Saharan African population. We collected data from outpatients presenting at two primary healthcare facilities in Blantyre, Malawi, from November 2020 to March 2022. Eligible participants were aged >1month old, with signs suggestive of COVID-19, and those not suspected of COVID-19, from whom we collected nasopharyngeal swabs for SARS-CoV-2 PCR testing, and sequenced positive samples to identify infecting-variants. In addition, we calculated the risk of presenting with a given symptom in individuals testing SARS-CoV-2 PCR positive before and during the Omicron variant-dominated period. Among 5176 participants, 6.4% were under 5, and 77% were aged 18 to 50 years. SARS-CoV-2 infection prevalence peaked in January 2021 (Beta), July 2021 (Delta), and December 2021 (Omicron). We found that cough (risk ratio (RR), 1.50; 95% confidence interval (CI), 1.00 to 2.30), fatigue (RR 2.27; 95% CI, 1.29 to 3.86) and headache (RR 1.64; 95% CI, 1.15 to 2.34) were associated with a high risk of SARS-CoV-2 infection during the pre-Omicron period. In comparison, only headache (RR 1.41; 95% CI, 1.07 to 1.86) did associate with a high risk of SARS-CoV-2 infection during the Omicron-dominated period. In conclusion, clinical symptoms associated with Omicron infection differed from prior variants and were harder to identify clinically with current symptom guidelines. Our findings encourage regular review of case definitions and testing policies to ensure case ascertainment.

## Introduction

As of June 2022, the COVID-19 pandemic has resulted in 543 million cases and 6.3 million deaths globally [1]. In sub-Saharan Africa, the pandemic has however been associated with a lower rate of hospitalisation and deaths than in Europe and the Americas [1], despite widespread SARS-CoV-2 community transmission [2], and low COVID-19 vaccine coverage [3]. Due to the continued emergence of SARS-CoV-2 variants of concern, surveillance is essential for monitoring the pandemic and informing public health interventions, however the optimal approach to surveillance in low-income, resource-poor settings is unclear [4].

By June 2022, Malawi had experienced four epidemic waves peaking in July 2020, January 2021, July 2021, and December 2021. There were 86,348 confirmed SARS-CoV-2 cases nationally, with 2,645 COVID-19-associated deaths [1]. However, due to the availability of testing there is considerable case under ascertainment, as evidence by the high seroprevalence of >65% observed in Malawi as of July 2021 [5]. Blantyre has had the highest number of reported COVID-19 cases in Malawi, with 28.6% of the national cases [6]. Recently, the Omicron variant has resulted in less hospitalisations and mortality in Malawi compared to the Delta variant [1], which has coincided with high seroprevalence of SARS-CoV-2 antibodies in Malawi and across sub-Saharan Africa [5, 7]. Further, Malawi introduced COVID-19 vaccines in March 2021 [8]. The COVID-19 vaccination coverage for Malawi is 4.5%, including the AstraZeneca, Janssen and Pfizer vaccines, with AstraZeneca vaccine constituting most of the doses [8].

Using data from early in the pandemic, a standardised case definition for COVID-19 was developed by the World Health Organisation (WHO) [9] and United States Centre for Disease Control (US CDC) [10], and these have allowed targeted SARS-CoV-2 testing. However, in sub-Saharan Africa, there is a high burden of other febrile illnesses such as malaria, pneumonia, TB and salmonellosis that have clinical features that overlap with COVID-19 [11, 12]. Further, there is limited data on the differences in clinical presentation between infections caused by different variants of concern (VOC), especially amongst non-hospitalised patients. To address these gaps, our study measured the prevalence of PCR-confirmed SARS-CoV-2 infection among outpatients presenting with medical conditions at primary healthcare facilities and compared the symptom profiles between Omicron and pre-Omicron infections.

## Methods

### Study design and population

From November 2020 to March 2022, we conducted a SARS-CoV-2 prevalence study in primary healthcare facilities in the city of Blantyre, southern Malawi. Blantyre is Malawi’s commercial city with a population of 800,264 (pop density, 3334/km^2^). Adults and children were recruited voluntarily from two government-owned primary healthcare facilities, Ndirande Health Centre and Limbe Health Centre, both overseen by the Blantyre District Health Office. Census data shows Ndirande HC serves a catchment area of 135,736, while Limbe HC serves a catchment area of 145,604, but the actual catchment is likely much higher.

From November 2020 to July 2021, individuals with medical conditions were screened at the facility’s outpatient services department. Following assessment by a clinician and a review of the individual’s health passport (patient retained medical record), a nasopharyngeal swab was collected for SARS-CoV-2 screening by SARS-CoV-2 by RT-PCR from patients suspected of COVID-19 according to the WHO case definition [9]. From August 2021, following a protocol amendment to include capturing a more detailed clinical history using the ISARIC symptom list [13–15], participants included both those suspected and those not suspected of COVID-19 according to the WHO case definition, with a 2:1 numerical bias towards those with suspected COVID-19. Patients with suspected COVID-19 were recruited as and when they presented to the facility, while those with not clinically suspected of COVID-19 were selected by approaching every third patient in health facility’s triage area.

### Data and specimen collection

Following informed consent and assent (for children) from August 2021, we used an abridged International Severe Acute Respiratory and Emerging Infection Consortium (ISARIC) Clinical Characterisation Protocol (CCP) electronic case report form (eCRF) [13–15] to collect demographic and clinical data from all participants. Study nurses collected nasopharyngeal swabs in Universal Transport Medium (UTM) (Copan, Brescia, Italy) from all participants. Samples were initially stored and transported to the Malawi-Liverpool-Wellcome Programme (MLW) laboratory on ice and processed within 48 hours.

### Laboratory testing

Nasopharyngeal swabs were tested for SARS-CoV-2 RNA using the CDC 2019-nCoV RNA RT-PCR diagnostic panel (Integrated DNA Technologies, Iowa, USA). A cycle threshold (Ct) value of <40 was considered positive for SARS-CoV-2 using QuantStudio Real-Time PCR software v1.3 (Applied Biosystems, UK). Ribonuclease protein was used as an internal control to identify presence of human RNA. A negative extraction control and a PCR no-template control were also performed with every test. The results of patients with positive PCR tests were shared with the Blantyre District Health Office for further follow up and patient management.

### Genomic sequencing and analysis

Samples were extracted using the Qiasymphony-DSP mini kit 200 (Qiagen, UK) with offboard lysis. Samples were then tested using the CDC N1 assay to confirm the Ct values before sequencing. Samples with a Ct value <27 were sequenced. The following sequencing protocols were used; ARTICv2 and v3 was used from November 2020 from July 2021 to July 2021 [16] and UNZA [17] from August 2021 on wards. Initially two primer pools were used, however a third pool was made for primer pairs that commonly had lower depth compared to the average [15]. PCR cycling conditions were adapted to the new sequencing primers, with annealing temperature changed to 60°C. Sequencing was carried out with the Oxford Nanopore Technologies MinION sequencer. Samples that had poor coverage (<70%) with the ARTIC primer set were repeated with the UNZA primer set.

For analysis of sequencing data, the lineage of each consensus genome was identified using pangolin with the following versions; pangolin v3.1.17, pangolearn 2021-12-06, constellations v0.1.1, scorpio v0.3.16, pango-designation used by pangoLEARN/Usher v1.2.105, pango-designation aliases v1.2.122 [18]. Samples were re-analysed when the Pangolin database was updated. The run was repeated if there was contamination in the negative control.

### Statistical analysis

We performed statistical analyses and graphical presentation using R statistical package, version 4.1.0. Categorical variables were summarized using frequency distributions and compared using Pearson’s Chi-squared test and Fisher’s exact test. The continuous variables were presented as median with interquartile range.

We employed multivariable logistic regression models, as implemented in the R package stats (version 3.6.2), to investigate odds of presenting with particular symptoms in Omicron compared pre-Omicron phases, adjusting for age and sex. A multivariable logistic regression model adjusting for age, sex, vaccination status, vaccination doses and days since last vaccine dose was also employed to investigate the impact of COVID-19 vaccination on PCR-confirmed SARS-CoV-2. P-values <0.05 were considered significant.

### Ethics approval

The study was approved by the College of Medicine Research and Ethics Committee (COMREC P.08/20/3099) and Liverpool School of Tropical Medicine Research Ethics Committee (LSTMREC 21–058). Written informed consent was obtained from the parent/guardian of each participant under 18 years of age, and from individual adult participants.

## Results

### Demographic and clinical characteristics of participants

From November 2020 through March 2022, 6147 (Ndirande Health Centre, n = 2899; Limbe Health Centre, n = 3248) individuals were approached, and 5188 participants were enrolled but 12 were excluded as they did not have sex recorded. Refusals were mostly from parents or guardians who did not consent for their children to undergo nasopharyngeal swabbing, and this did not change throughout the study period. Overall, the participants’ median age was 28 years (IQR 21–38), of which 6.4% (331/5176) were under 5 years, 9.2% (331/5176) were 6 to 17 years (479/5176), 77% (4000/5176) were 18 to 50 years, and 7.1% (368/5176) were above 50 years old. Of the total 50% (2596/5176) were female (**Table 1**).

**Table 1:** Participant characteristics for main cohort.

Characteristic	N = 5,176^1^
**Sex**	
Female	2,596 (50%)
Male	2,580 (50%)
**Age**	28 (21, 38)
**Age group**	
0–5 years	331 (6.4%)
6–12 years	228 (4.4%)
13–17 years	249 (4.8%)
18–50 years	4,000 (77%)
51+ years	368 (7.1%)
**Sars-coV-2 PCR positivity**	
Negative	3,992 (77%)
Positive	1,184 (23%)

^1^N (%); Median (IQR)

### Prevalence of SARS-CoV-2 infection and genomic surveillance

**Fig 1:**
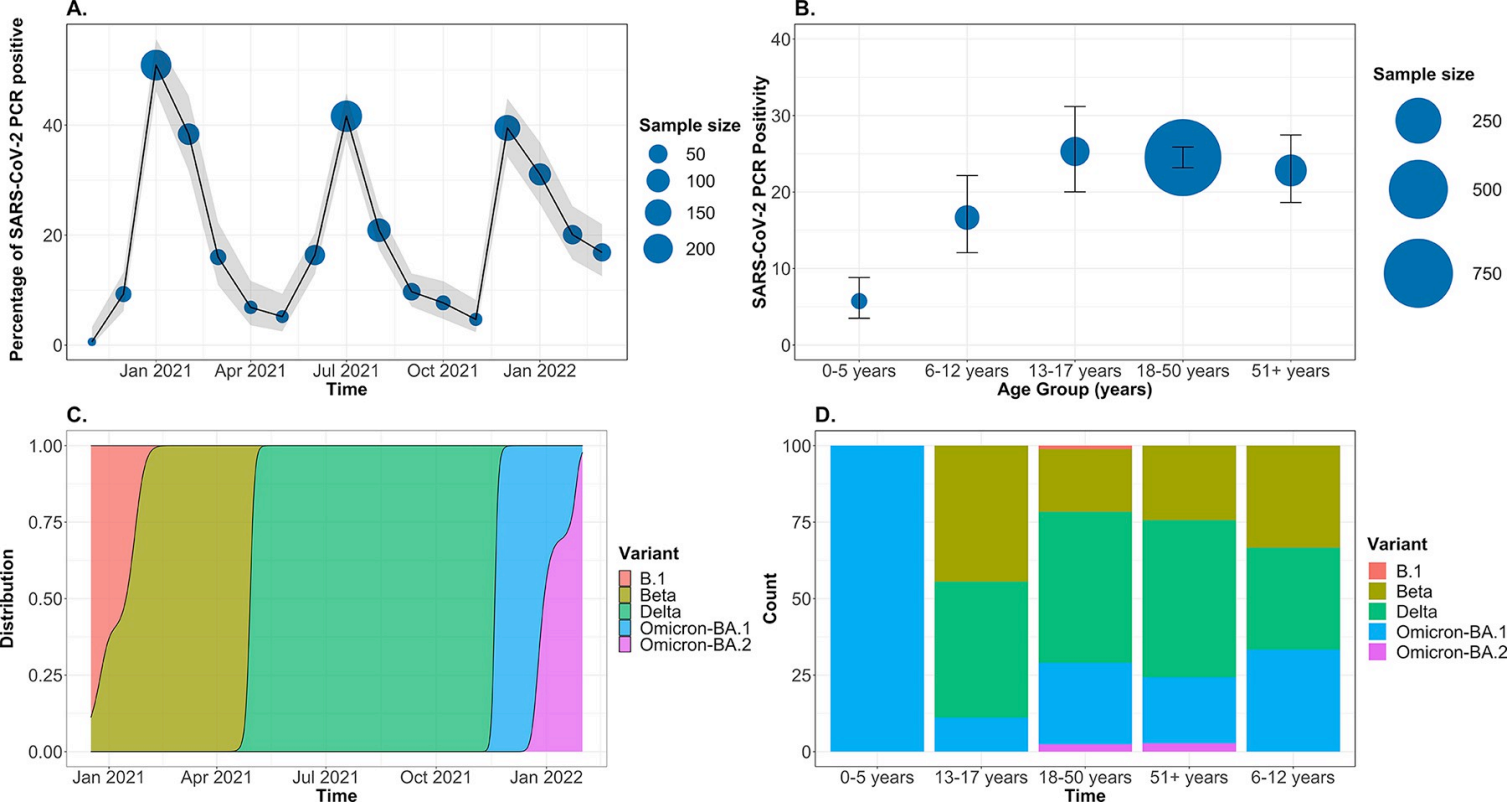
Prevalence of SARS-CoV-2 infections. A) Prevalence of SARS-CoV-2 infections across age groups. B) Prevalence of SARS-CoV-2 infections over time. Grey area represents confidence intervals. C) SARS-CoV-2 variants of concern across the three pandemic waves. D) SARS-CoV-2 variants of concern across age groups. (n = 402).

The overall prevalence of PCR-confirmed SARS-CoV-2 infection was 23% (1187/5176) (**Table 1**). SARS-CoV-2 prevalence varied over time, with three distinct peaks over the study period, namely January 2021, July 2021, and December 2021 (**Fig 1A**). SARS-CoV-2 prevalence was lowest in those under 5 years of age (5.74% [CI 3.49–8.82]) compared to all other age groups (6-12yrs, 16.7% [12.1–22.2]; 13-17yrs, 25.3% [20.0–31.2]; 18-50yrs, 24.5% [23.2–25.9]; 50+yrs, 22.8% [18.6–27.5]) (**Fig 1B**). The three prevalence peaks corresponded with emergence of variants of concern (VOC), including Beta (B.1.351; January 2021), Delta (1.617.2; July 2021) and Omicron (BA.1/2; December 2021) (**Fig 1C**). Only Omicron (BA.1) was detectable in all age groups (**Fig 1D**).

### Symptoms associated with SARS-CoV-2 infection, pre- and during the Omicron-dominated phase

Forty-nine percent (2520/5176) of the total participants were recruited from August 2021 to March 2022, hence had detailed symptom and medical history (**S1 Fig**). Out of these 2509 had complete symptomology data and were used in the subsequent analysis. Using a multivariable analysis, cough (71% vs 68%, risk ratio, 1.50; 95% CI, 1.00 to 2.30, p = 0.056), fatigue (14% vs 6.3%, risk ratio, 2.27; 95% CI, 1.29 to 3.86, p = 0.003) and headache (49% vs 37%, risk ratio, 1.64; 95% CI, 1.15 to 2.34, p = 0.007) were associated with a high risk of PCR-confirmed SARS-CoV-2 infection during the pre-Omicron period (**Table 2 and S1 Table**). While, during the Omicron-dominated period, only headache (39% vs 30%, risk ratio, 1.41; 95% CI, 1.07 to 1.86, p = 0.015) was associated with a high risk of PCR-confirmed SARS-CoV-2 infection.

**Table 2:** Symptoms associated with SARS-CoV-2 infection.

Characteristic	Univariable (Pre-Omicron)				Multivariable (Pre-Omicron)			Univariable (Omicron)				Multivariable (Omicron)		
	N	OR^*1*^	95% CI^*1*^	p-value	OR^*1*^	95% CI^*1*^	p-value	N	OR^*1*^	95% CI^*1*^	p-value	OR^*1*^	95% CI^*1*^	p-value
**Fever**	1,311							1,198						
No		Ref.	—		Ref.	—			Ref.	—		Ref.	—	
Yes		0.76	0.46, 1.21	0.3	0.65	0.39, 1.05	0.093		1.30	0.94, 1.78	0.11	1.23	0.88, 1.71	0.2
**Cough**	1,311							1,198						
No		Ref.	—		Ref.	—			Ref.	—		Ref.	—	
Yes		1.15	0.80, 1.68	0.5	1.50	1.00, 2.30	**0.056**		0.97	0.75, 1.25	0.8	1.04	0.79, 1.38	0.8
**Fatigue**	1,311							1,198						
No		Ref.	—		Ref.	—			Ref.	—		Ref.	—	
Yes		2.34	1.37, 3.87	**0.001**	2.27	1.29, 3.86	**0.003**		1.40	0.65, 2.89	0.4	1.24	0.55, 2.64	0.6
**Loss of smell**	1,311							1,198						
No		Ref.	—		Ref.	—			Ref.	—		Ref.	—	
Yes		1.29	0.59, 2.55	0.5	0.89	0.34, 2.16	0.8		1.51	0.63, 3.38	0.3	0.70	0.22, 2.06	0.5
**Loss of taste**	1,311							1,198						
No		Ref.	—		Ref.	—			Ref.	—		Ref.	—	
Yes		1.80	0.99, 3.11	**0.044**	1.79	0.83, 3.65	0.12		2.07	1.05, 4.01	**0.031**	2.04	0.81, 5.13	0.12
**Sore throat**	1,311							1,198						
No		Ref.	—		Ref.	—			Ref.	—		Ref.	—	
Yes		1.14	0.67, 1.85	0.6	1.03	0.59, 1.72	>0.9		1.36	0.95, 1.93	0.091	1.35	0.93, 1.93	0.11
**Headache**	1,311							1,198						
No		Ref.	—		Ref.	—			Ref.	—		Ref.	—	
Yes		1.63	1.16, 2.28	**0.005**	1.64	1.15, 2.34	**0.007**		1.49	1.14, 1.95	**0.003**	1.41	1.07, 1.86	**0.015**
**Muscle ache**	1,311							1,198						
No		Ref.	—		Ref.	—			Ref.	—		Ref.	—	
Yes		1.25	0.83, 1.85	0.3	1.15	0.72, 1.80	0.6		1.33	1.01, 1.74	**0.044**	1.28	0.94, 1.73	0.12
**Joint pain**	1,311							1,198						
No		Ref.	—		Ref.	—			Ref.	—		Ref.	—	
Yes		1.39	0.77, 2.38	0.2	1.07	0.54, 2.01	0.8		1.44	0.87, 2.34	0.15	1.00	0.57, 1.73	>0.9
**Abdominal pain**	1,311							1,198						
No		Ref.	—		Ref.	—			Ref.	—		Ref.	—	
Yes		1.24	0.75, 1.98	0.4	1.33	0.76, 2.23	0.3		1.10	0.69, 1.72	0.7	1.29	0.79, 2.08	0.3
**Diarrhoea**	1,311							1,198						
No		Ref.	—		Ref.	—			Ref.	—		Ref.	—	
Yes		0.80	0.39, 1.51	0.5	0.89	0.41, 1.78	0.8		0.79	0.45, 1.31	0.4	0.87	0.49, 1.49	0.6
**Shortness of breath**	1,311							1,198						
No		Ref.	—		Ref.	—			Ref.	—		Ref.	—	
Yes		0.58	0.25, 1.14	0.15	0.55	0.23, 1.15	0.14		0.79	0.46, 1.30	0.4	0.80	0.41, 1.52	0.5
**Chest pain**	1,311							1,198						
No		Ref.	—		Ref.	—			Ref.	—		Ref.	—	
Yes		0.92	0.58, 1.41	0.7	0.83	0.51, 1.32	0.5		1.21	0.83, 1.74	0.3	1.32	0.90, 1.93	0.2
**Runny nose**	1,311							1,198						
No		Ref.	—		Ref.	—			Ref.	—		Ref.	—	
Yes		0.84	0.58, 1.21	0.4	0.78	0.52, 1.16	0.2		0.96	0.70, 1.31	0.8	0.96	0.69, 1.32	0.8
**Pneumonia**	1,311							1,198						
No		Ref.	—		Ref.	—			Ref.	—		Ref.	—	
Yes		1.08	0.51, 2.05	0.8	1.51	0.67, 3.13	0.3		0.68	0.37, 1.19	0.2	0.78	0.37, 1.58	0.5

^*1*^ OR = Odds Ratio, CI = Confidence Interval

### Impact of COVID-19 vaccination on the risk of PCR-confirmed SARS-CoV-2 infection

Eighty percent (2009/2520) of the participants with detailed symptomology were eligible for vaccination (18 years and above) and had complete vaccination history. We, therefore, used these individuals to determine whether the risk of PCR-confirmed SARS-CoV-2 infection was different between vaccinated compared to unvaccinated adults. Using a multivariable analysis, adjusting for days since last vaccine dose, sex, age and recruitment period; COVID-19 vaccination was not associated with a reduced risk of PCR-confirmed SARS-CoV-2 infection (1 dose, OR 1.10[CI 0.39–2.66]; 2 doses, OR 1.11[CI 0.40–2.57]; <91 days, OR 1.93[CI 0.74–5.68]; 91+ days, OR 1.54[CI 0.63–4.34]) (**Table 3**). However, the Omicron recruitment period (December 2021 to March 2022), was associated with a threefold increase in the risk of PCR-confirmed SARS-CoV-2 infection than the pre-Omicron period (August 2021 to November 2021) (OR 2.56 [CI 2.02–3.26]) (**Table 3**).

**Table 3:** Participant characteristics for PCR-confirmed SARS-CoV-2 infected adult patients.

Characteristic	Overall, N = 447^*1*^	Omicron phase, N = 302^*1*^	Pre-omicron phase, N = 145^*1*^	p-value^*2*^
**Sex**				0.2
Female	232 (52%)	151 (50%)	81 (56%)	
Male	215 (48%)	151 (50%)	64 (44%)	
**Age**	31 (24, 39)	31 (25, 38)	32 (23, 39)	0.8
**HIV status**				0.4
Negative	277 (84%)	173 (83%)	104 (87%)	
Positive	51 (16%)	35 (17%)	16 (13%)	
Unknown	119	94	25	
**PLHIV on ART**	49 (11%)	34 (11%)	15 (10%)	0.8
**Hypertension**	7 (1.6%)	5 (1.7%)	2 (1.4%)	>0.9
**Diabetes**	4 (0.9%)	0 (0%)	4 (2.8%)	**0.011**
**Asthma**	15 (3.4%)	8 (2.6%)	7 (4.8%)	0.3
**COVID-19 vaccination (doses)**				**0.025**
0	310 (69%)	206 (68%)	104 (72%)	
1	63 (14%)	37 (12%)	26 (18%)	
2	74 (17%)	59 (20%)	15 (10%)	
**Days since last vaccine dose**	137 (51, 187)	173 (141, 208)	78 (40, 137)	**<0.001**
Unknown	375	263	112	

^*1*^ n (%); Median (IQR)

^*2*^ Pearson’s Chi-squared test

Wilcoxon rank sum test; Fisher’s exact test

### Symptoms associated with PCR-confirmed SARS-CoV-2 infection change with variants

Based on the genomic surveillance data (**Fig 1C**), we assigned all individuals infected within the period from August 2021 to November 2021 as pre-Omicron infections (most commonly Delta infections), and those from December 2021 to March 2022 as Omicron infections. Twenty-two percent (447/2009) of vaccine-eligible patients who had provided the date since the last vaccine dose had PCR-confirmed SARS-CoV-2 infection (**Table 4**). Thirty-five out of 228 patients were less than 18 years of age and had PCR-confirmed SARS-CoV-2 infection (**S2 Table**). As such subsequent analyses only focused on the adult population (≥18 years). Fifty percent (151/302) of these patients in Omicron phase were female, while 56% (81/145) were female in the pre-Omicron phase. The median age of patients from the two phases was similar (31 years [IQR 25–38] vs. 32[IQR 23–39], p = 0.8) (**Table 4**). Among those with known self-reported HIV status, the HIV prevalence was 16%, which was similar between the two phases (17% vs 13%, p = 0.4). Of those People Living with HIV (PLHIV), 97% (34/35) in the Omicron phase and 94% (15/16) in the pre-Omicron phase were on antiretroviral therapy. Other chronic illnesses were uncommon, with only 1.6% (7/447), 0.9% (4/447) and 3.4% (15/447) self-reported to have hypertension, diabetes, and asthma, respectively, although hypertension and diabetes, in particular, are widely underdiagnosed in Malawi [19, 20]. Thirty-one percent (137/447) of the participants reported being vaccinated with at least one dose of the COVID-19 vaccine (AstraZeneca vaccine (80% (109/137)) or Janssen vaccine (20% (28/137)). Of which, 54% (74/447) had self-reported to have received at least two doses of the AstraZeneca vaccine, with more patients in the Omicron phase than the pre-Omicron phase (20% (59/302) vs. 10% (15/145), p = 0.025). Moreover, the days since last vaccine dose were longer in the Omicron phase than pre-Omicron phase (173[IQR 141–208] vs. 78[IQR 40–137], p<0.001).

**Table 4:** COVID-19 vaccination and the risk of PCR-confirmed SARS-CoV-2 infection.

Characteristic	PCR positivity				Univariable				Multivariable		
	Overall, N = 2,009^*1*^	Negative, N = 1,627^*1*^	Positive, N = 382^*1*^	p-value^*2*^	N	OR^*3*^	95% CI^*3*^	p-value	OR^*3*^	95% CI^*3*^	p-value
**COVID-19 vaccine doses**				**0.027**	2,009						
0	1,718 (100%)	1,408 (82%)	310 (18%)			Ref.	—		Ref.	—	
1	142 (100%)	107 (75%)	35 (25%)			1.49	0.98, 2.20	**0.053**	1.10	0.39, 2.66	0.8
2	149 (100%)	112 (75%)	37 (25%)			1.50	1.00, 2.20	**0.042**	1.11	0.40, 2.57	0.8
**Days since last vaccine dose**				**0.003**	2,009						
0	1,763 (100%)	1,447 (82%)	316 (18%)			Ref.	—		Ref.	—	
<91 days	90 (100%)	68 (76%)	22 (24%)			1.48	0.88, 2.39	0.12	1.93	0.74, 5.68	0.2
91+ days	156 (100%)	112 (72%)	44 (28%)			1.80	1.23, 2.58	**0.002**	1.54	0.63, 4.34	0.4
**Sex**				0.4	2,009						
Female	1,001 (100%)	803 (80%)	198 (20%)			Ref.	—		Ref.	—	
Male	1,008 (100%)	824 (82%)	184 (18%)			0.91	0.72, 1.13	0.4	0.84	0.67, 1.06	0.15
**Age group**				0.13	2,009						
18–50 years	1,904 (100%)	1,536 (81%)	368 (19%)			Ref.	—		Ref.	—	
51+ years	105 (100%)	91 (87%)	14 (13%)			0.64	0.35, 1.10	0.13	0.62	0.33, 1.09	0.12
**Recruitment period**				**<0.001**	2,009						
Pre-omicron phase	1,080 (100%)	943 (87%)	137 (13%)			Ref.	—		Ref.	—	
Omicron phase	929 (100%)	684 (74%)	245 (26%)			2.47	1.96, 3.11	**<0.001**	2.56	2.02, 3.26	**<0.001**

^*1*^ n (%)^*2*^

Pearson’s Chi-squared test ^*3*^

OR = Odds Ratio, CI = Confidence Interval

Clinical symptoms associated with PCR-confirmed SARS-CoV-2 infections were different during the Omicron and pre-Omicron phases (**S3 Table**). Cough (70% vs. 54%, p<0.001), fatigue (14% vs 3.6%, p<0.001), loss of taste (10% vs. 5.0%, p = 0.033), headache (50% vs. 39%, p = 0.034), and abdominal pain (14% vs. 8.3%, p = 0.043) were more frequent in patients from the pre-Omicron phase compared to the Omicron phase. In contrast, muscle ache was more common in the Omicron phase than pre-Omicron phase (35% vs 25%, p = 0.029).

**Fig 2:**
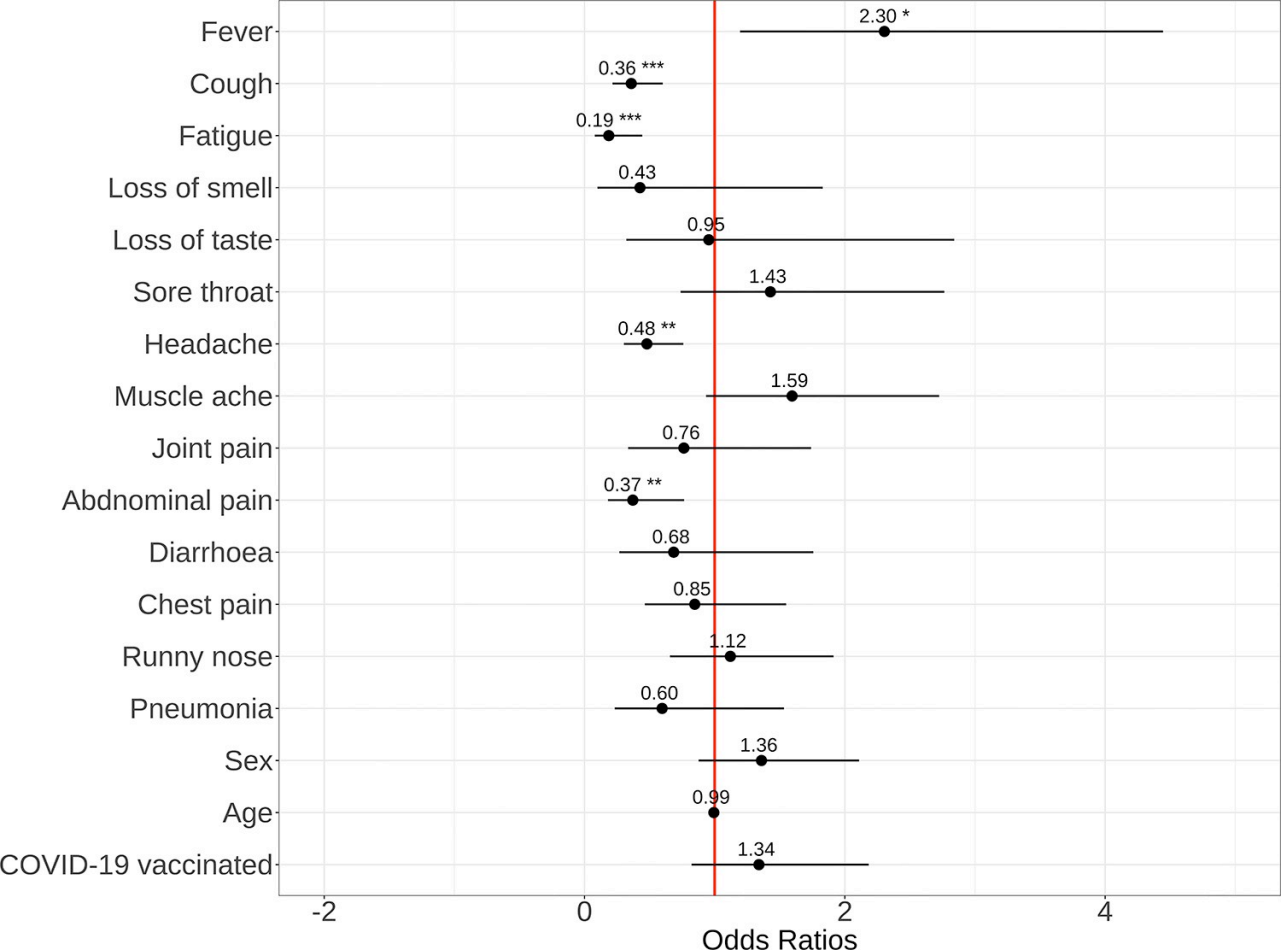
Clinical symptoms associated with PCR-confirmed SARS-CoV-2 infection. Differences in clinical symptoms of PCR-confirmed SARS-CoV-2 infected adults during the Omicron and Pre-Omicron phases (n = 447).

Controlling for age, sex and COVID-19 vaccination status in a multivariable analysis, cough (OR 0.37 [CI 0.22–0.61], p<0.001), fatigue (OR 0.20 [CI 0.08–0.48], p<0.001), headache (OR 0.47 [CI 0.29–0.79], p = 0.001), and abdominal pain (0R 0.38 [CI 0.18–0.78]) were independently associated with pre-Omicron than Omicron SARS-CoV-2 PCR positive diagnosis (**Fig 2 and Table 5).** Conversely, fever was independently associated with Omicron than pre-Omicron SARS-CoV-2 PCR positive diagnosis (OR 2.46 [CI 1.29–4.97]) (**Fig 2 and Table 5)**.

**Table 5:** Prevalence of symptoms among PCR-confirmed SARS-CoV-2 infected adult patients during pre-Omicron and Omicron periods.

Characteristic	Univariable				Multivariable		
	N	OR^*1*^	95% CI^*1*^	p-value	OR^*1*^	95% CI^*1*^	p-value
**Fever**	447						
No		Ref.	—		Ref.	—	
Yes		1.58	0.91, 2.82	0.11	2.33	1.23, 4.66	**0.012**
**Cough**	447						
No		Ref.	—		Ref.	—	
Yes		0.49	0.32, 0.74	**<0.001**	0.36	0.21, 0.60	**<0.001**
**Fatigue**	447						
No		Ref.	—		Ref.	—	
Yes		0.22	0.10, 0.47	**<0.001**	0.18	0.07, 0.41	**<0.001**
**Loss of smell**	447						
No		Ref.	—		Ref.	—	
Yes		0.47	0.17, 1.29	0.13	0.45	0.10, 1.97	0.3
**Loss of taste**	447						
No		Ref.	—		Ref.	—	
Yes		0.45	0.21, 0.96	**0.037**	0.81	0.27, 2.51	0.7
**Sore throat**	447						
No		Ref.	—		Ref.	—	
Yes		1.39	0.78, 2.58	0.3	1.44	0.75, 2.86	0.3
**Headache**	447						
No		Ref.	—		Ref.	—	
Yes		0.65	0.44, 0.97	**0.035**	0.47	0.29, 0.74	**0.001**
**Muscle ache**	447						
No		Ref.	—		Ref.	—	
Yes		1.64	1.06, 2.58	**0.030**	1.59	0.94, 2.76	0.089
**Joint pain**	447						
No		Ref.	—		Ref.	—	
Yes		0.76	0.40, 1.49	0.4	0.69	0.30, 1.61	0.4
**Abdominal pain**	447						
No		Ref.	—		Ref.	—	
Yes		0.53	0.29, 1.00	**0.046**	0.37	0.18, 0.76	**0.007**
**Diarrhoea**	447						
No		Ref.	—		Ref.	—	
Yes		0.66	0.29, 1.56	0.3	0.69	0.27, 1.82	0.4
**Shortness of breath**	447						
No		Ref.	—		Ref.	—	
Yes		1.21	0.54, 2.99	0.7	2.65	0.88, 8.76	0.094
**Chest pain**	447						
No		Ref.	—		Ref.	—	
Yes		0.79	0.47, 1.34	0.4	0.83	0.46, 1.53	0.5
**Runny nose**	447						
No		Ref.	—		Ref.	—	
Yes		0.75	0.47, 1.18	0.2	1.10	0.65, 1.90	0.7
**Pneumonia**	447						
No		Ref.	—		Ref.	—	
Yes		0.79	0.34, 1.92	0.6	0.35	0.11, 1.10	0.071
**Sex**	447						
Female		Ref.	—		Ref.	—	
Male		1.27	0.85, 1.89	0.2	1.38	0.89, 2.16	0.15
**Age**	447	1.00	0.98, 1.02	0.7	0.99	0.97, 1.02	0.5
**COVID-19 vaccinated**	447						
No		Ref.	—		Ref.	—	
Yes		1.18	0.77, 1.84	0.5	1.35	0.83, 2.22	0.2

^*1*^ OR = Odds Ratio

CI = Confidence Interval

## Discussion

In a setting where there is a high burden of presentations with infectious disease, we found that the symptoms associated with SARS-CoV-2 Omicron infection have become considerably less distinct, differing significantly from those infections with pre-Omicron variants (predominantly Delta). Indeed fever, which is common to many infectious presentations [11, 12], was most prevalent among presumed Omicron infected patients, while headache, cough, fatigue, and abdominal pain were significantly more prevalent among pre-Omicron cases.

Our data showing a different symptom profile associated with Omicron infection is consistent with studies elsewhere [21] with headache being prominent in three other studies from the UK [21–23]. However, we observed high odds for presenting with fever in presumed Omicron-infected patients than pre-Omicron patients, in contrast with the two studies in the UK [21, 23]. The main differences between the Malawi study and the UK studies are age and prevalence of Omicron sub-lineages, with Malawi cohort being a younger population and having predominantly BA.1 at time of sampling. BA.1 is associated with a different symptom profile than BA.2 [22]. Together, our findings and those of others suggest that the clinical case definition of COVID-19 used for testing and surveillance may need to be revised to maintain case ascertainment.

Data from Malawi and elsewhere has shown that the Omicron variant has presented with less severe disease, hospitalisation and deaths, than the pre-Omicron VOCs [1, 24]. The Omicron variant have been shown to be less capable of transition from the upper to lower respiratory tract infection [25], and this could potentially contribute to the low incidence of severe disease. However, data in non-immunised populations in Hong Kong indicate that Omicron is not intrinsically mild [26, 27], suggesting that immune response or past exposure could be an important determinant of this low severity. Data from South Africa has shown that high SARS-CoV-2 seroprevalence has been associated with low number of deaths and hospitalisation attributed to the Omicron variant [24]. In Malawi, seroprevalence data has shown that more than 70% of the population had anti-SARS-CoV-2 receptor binding domain (RBD) antibodies before the Omicron variant pandemic wave [5]. In line with previous findings [28], COVID-19 vaccination was not associated with a reduced risk of PCR-confirmed SARS-CoV-2 infection, especially during the Omicron wave. It is therefore plausible that the altered clinical presentation observed in our study could also be attributed to pre-existing immunity from prior SARS-CoV-2 exposure.

Furthermore, our findings align with the temporal dynamics of the COVID-19 pandemic waves in Malawi and the region [1, 5, 24]. A high SARS-CoV-2 prevalence of 30–50% among patients presenting to primary healthcare at the peak of the three pandemic waves, is consistent with high reported national COVID-19 cases during the same period [1]. Furthermore, consistent with genomic surveillance [15, 29], our sentinel surveillance correctly identified the VOCs driving the local pandemic waves. Due to the consistency in our sampling over time, we were able to provide real-time data to aid public health response in Malawi, especially on the identification of VOCs driving community transmission. Collectively, this indicates that sentinel surveillance backed up by diagnostics and genomics data could be an early warning system for national pandemic response in resource-limited settings, considering that by the time hospitalisations are rising it is already too late to intervene effectively.

Our study had several limitations. Firstly, the study was conducted in urban Blantyre and findings may not be generalisable to rural settings. Secondly, our study cohort (median age 28 years (IQR 21–38)) was not fully representative of the population structure in Malawi (median age 17.5 years) [30]. Thirdly, since our genomic surveillance was limited to a subset of samples with low PCR CT values, this approach biases our identification of variants to those causing high viral burden infections at time of recruitment. Lastly, our analysis of the impact of COVID-19 vaccination did not adjust for immunity induced following previous exposure to SARS-CoV-2, as it has been previously shown to be protective against symptomatic COVID-19 [31, 32]. The SARS-CoV-2 seroprevalence has been reported to be very high in Malawi [5], despite a low vaccination coverage [33].

In conclusion, our study demonstrates changes in clinical symptoms overtime, aligned to infecting variant, indicating that case definitions of COVID-19 need constant monitoring and revision to match SARS-CoV-2 evolution to maintain its relevance for institutional and national testing policies. This study also highlights the importance and utility of sentinel surveillance in low-resourced settings to aid timely public health response against the COVID-19 pandemic and future pandemics.

## Supporting information

S1 Table Clinical symptom presentation during the pre-Omicron and Omicron period. (XLSX)

S2 Table Participant characteristics for PCR-confirmed SARS-CoV-2 infected under 18s during the pre-Omicron and Omicron period. (XLSX)

S3 Table Prevalence of symptoms among PCR-confirmed SARS-CoV-2 infected adult patients during pre-Omicron and Omicron period. (XLSX)

S1 Fig Flowchart of participant enrolment and testing in two primary healthcare facilities in Blantyre City, Malawi, November 2020 –March 2022. (TIFF)

S1 Data *Cocosuall*. The dataset contains the underlying data for Table 1.
(CSV)

S2 Data *Cocosuseq*. The dataset contains the underlying data for Fig 1.
(CSV)

S3 Data *Cocosusym*. The dataset contains the underlying data for Tables 2–5 and Fig 2.
(CSV)
