# Supplementary figures and images for: Different clinical features in Malawian outpatients presenting with COVID-19 prior to and during Omicron variant dominance: A prospective observational study
Source: PLOS Glob Public Health. 2023 Mar 8;3(3):e0001575. doi: 10.1371/journal.pgph.0001575 (PMC10022204; doi:10.1371/journal.pgph.0001575)

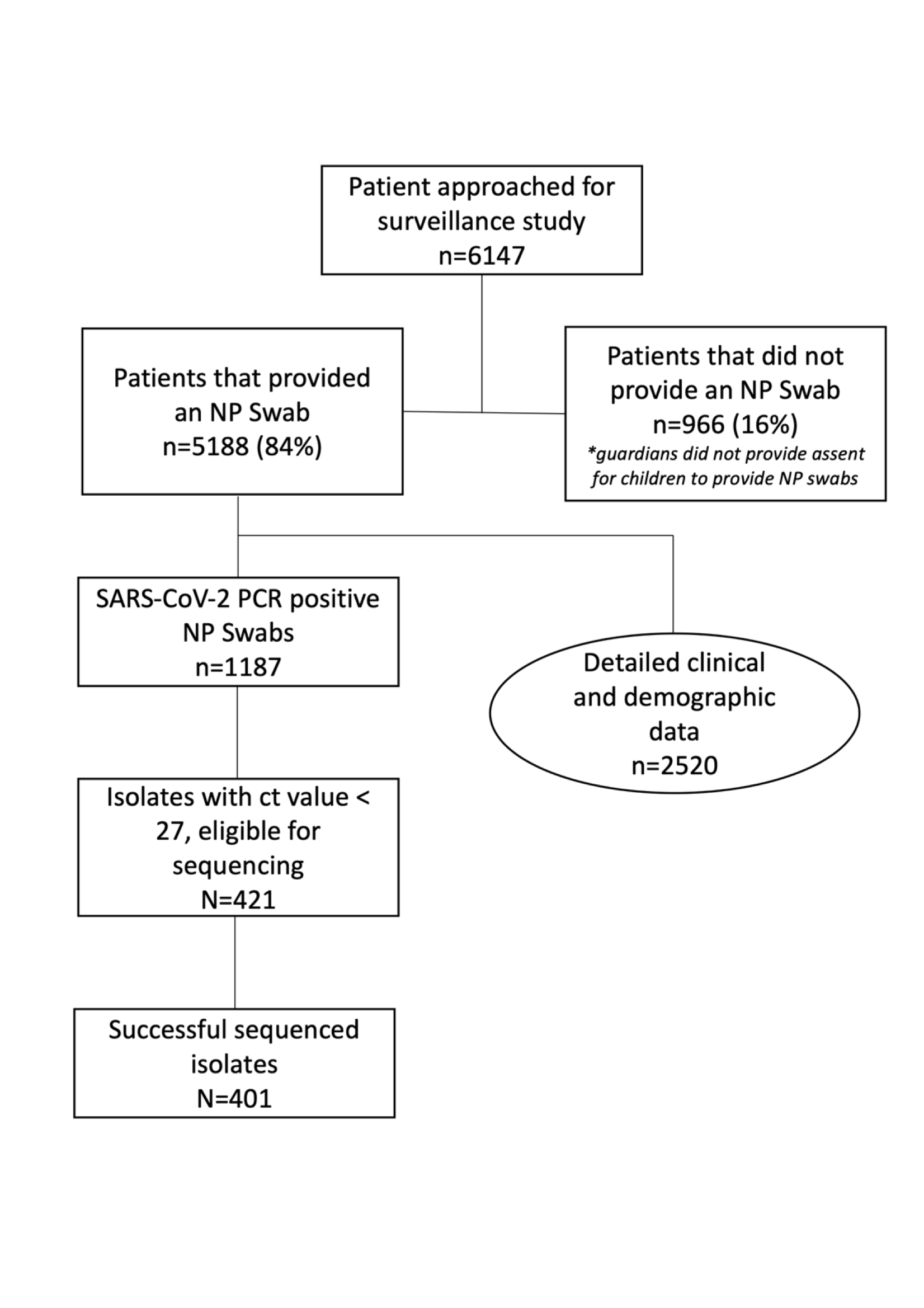

Supplement: S1 Fig — (TIFF) [file pgph.0001575.s004.tiff]
